# Genomic characterization and diversity of dsRNA viruses and their killer satellites in a natural population of the yeast *Saccharomyces paradoxus*

**DOI:** 10.1093/ve/veag043

**Published:** 2026-07-16

**Authors:** Mahsan Nematbakhsh, Vassiliki Koufopanou, Anthony Cass, Austin Burt

**Affiliations:** Department of Life Sciences, Imperial College London, Silwood Park Campus, Ascot SL5 7PY, United Kingdom; Department of Life Sciences, Imperial College London, Silwood Park Campus, Ascot SL5 7PY, United Kingdom; Department of Chemistry, Imperial College London, White City Campus, London W12 0BZ, United Kingdom; Department of Life Sciences, Imperial College London, Silwood Park Campus, Ascot SL5 7PY, United Kingdom

**Keywords:** *Saccharomyces paradoxus*, dsRNA virus, preprotoxin, killer toxin, 5′ upstream sequence, yeast

## Abstract

Killer yeasts secrete protein toxins that inhibit other yeast strains, a trait often encoded by M satellites of L-A dsRNA viruses. These systems serve as important molecular models, yet their adaptive significance in natural settings remains unclear. This study surveyed 60 strains from a natural *Saccharomyces paradoxus* population in the UK to characterize dsRNA viral genomic diversity. Our survey revealed 27% of strains showed killer activity mediated by dsRNA viruses. Additionally, five of 18 nonkiller strains also contained dsRNA viruses. Deep sequencing of pooled dsRNAs from 17 strains identified six distinct M satellite types, including three novel lineages, with single-nucleotide and structural polymorphisms creating multiple variant sequences within types. Predicted preprotoxin proteins generally contained post-translational modification sites necessary for toxin maturation, although with variable numbers among different types, potentially affecting processing and expression. Eight L-A virus sequences were also assembled. These were all closely related to each other and clustered phylogenetically with viruses from other European *S. paradoxus* strains. Extended 5′ sequence analysis revealed novel structural features in both L-As and their M satellites, including a pair of inverted repeats ending in a pair of inverted conserved motifs (GA_5–6_ and corresponding U_5–6_C in Ms and GAAUA and corresponding UAUUC in L-As), which is, in turn, flanked by a pair of direct repeats. The pair of conserved motifs also exists in all described *S. cerevisiae* M satellites. The observed diversity of dsRNA satellites within a single yeast population is a challenge to explain, with many evolutionary forces potentially contributing.

## Introduction

Strains of many yeast species have been shown to have so-called killer activity, producing a protein toxin that kills or inhibits the growth of some other yeasts, of the same and/or different species, but that they themselves are immune to (Woods and Bevan 1968, Magliani et al. 1997, Liu et al. 2015). The genetic basis of toxin production (and immunity) varies widely (Klassen et al. 2017, Schaffrath et al. 2018). In some cases, the toxin is encoded by a nuclear gene, while in others it is encoded by nonchromosomal elements, either dsRNA viruses and their satellites or dsDNA ‘virus-like elements’ (VLEs). In the case of the dsRNA viruses, there are two RNA components, an M satellite dsRNA that encodes the preprotoxin that is processed into the secreted killer (K) toxin, and an L-A helper virus that encodes the Gag and Pol proteins needed for capsid formation and RNA replication for both itself and the M satellite. The dsDNA VLEs also have a two-component organization (Magliani et al. 1997, Schmitt and Breinig 2006, Schaffrath et al. 2018).

**Figure 1:**
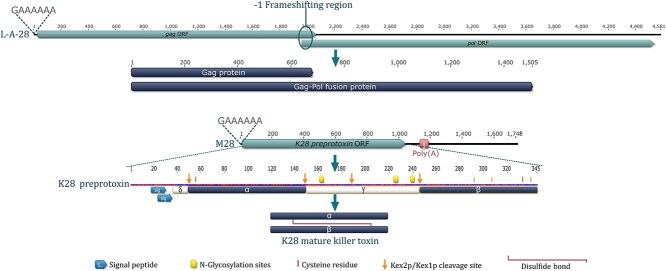
Structural organization of the L-A dsRNA virus, M satellite, and their encoded proteins. The L-A and M dsRNAs, as well as the killer preprotoxins of *S. cerevisiae* and *S. paradoxus*, share similar structural features. L-A-28 and M28 are shown as representative examples.

Several types of M satellites have been identified in two closely related species of *Saccharomyces, Saccharomyces cerevisiae* (M1, M2, and M-lus) and *S. paradoxus* [M62, M28, M74, M21, M66 (a close relative of M21), M45, and M1L (which shows homology to M1)]. These elements encode the killer toxins K1, K2, K-lus, K62, K28, K74, K21, K66, K45, and K1L, respectively. Despite the lack of sequence similarity among different M types, they all share a conserved structural organization: each M dsRNA begins with a conserved GA_5–6_ motif, followed by a coding region, a poly (A) tract, and a downstream noncoding region (Fig. 1) (Rodríguez-Cousiño et al. 2011, Rodríguez-Cousiño et al. 2017, Vepštaitė-Monstavičė et al. 2018, Fredericks et al. 2021a, Rodriguez-Cousiño et al. 2022). The coding region encodes a preprotoxin that is proteolytically processed to generate the functional toxin.

Almost all preprotoxins seem to share a similar structural organization, consisting of four subunits, δ, α, γ, and β (Fig. 1) (Riffer et al. 2002, Gier et al. 2020, Rodriguez-Cousiño et al. 2022, Creagh et al. 2025a). In the well-studied killer toxins, K1, K28, and K74, following translation, preprotoxins undergo a series of post-translational modifications in the endoplasmic reticulum (ER) and Golgi apparatus before being secreted into the extracellular environment as an active α/β heterodimer. All preprotoxins contain an N-terminal signal peptide that directs their entry into the ER. Processing in the ER and Golgi includes: (i) cleavage of the signal peptide (pre-region) by signal peptidase; (ii) N-glycosylation of the γ subunit; (iii) formation of a single disulfide bond between the α and β subunits mediated by protein disulfide isomerase; (iv) proteolytic cleavage by Kex2p/Kex1p at three sites to remove the δ (pre-region) and γ subunit; and (v) trimming of the C-termini of the α and β subunits by Kex1p (Fig. 1) (Riffer et al. 2002, Gier et al. 2020, Rodriguez-Cousiño et al. 2022, Molina-Vera et al. 2024). A recent study on K62 suggested that its preprotoxin has a different subunit organization to that of other killer toxins. Only a single Kex2p/Kex1p cleavage site was identified in the preprotoxin sequence, though its functionality remains uncertain. K62 probably has a structure similar to that of the aerolysin family proteins and can form homodimeric and homo-oligomeric structures following maturation (Creagh et al. 2025).

In contrast to M satellites, the sequences of L-A variants are closely related, sharing at least 73% nucleotide identity (Rodríguez-Cousiño et al. 2017). In *S. cerevisiae*, the conserved motif at the 5′ end of L-A (GA_5_) is identical to that of M satellites, thought to be part of the signal for transcription initiation (Rodríguez-Cousiño et al. 2011, Ramírez et al. 2021). The L-A genome contains two overlapping open reading frames (ORFs) that encode the Gag protein and a Gag–Pol fusion protein, the latter produced *via* a −1 ribosomal frameshift within the overlapping region (Fig. 1) (Wickner 1996a, Magliani et al. 1997).

In addition to L and M dsRNAs, several defective dsRNA species have been described, including X, S, and NS satellites, which are deletion derivatives of L-A, M1 and M2, respectively. These dsRNAs retain the nucleotide sequences required for replication and encapsidation but do not express any active protein and are considered defective interfering RNAs (Somers 1973, Vodkin et al. 1974, Sweeney et al. 1976, Bruenn and Kane 1978, Fried and Fink 1978, Bostian et al. 1980, Esteban and Wickner 1988, Cansado et al. 1999).

Unlike chromosomally-encoded toxin genes, which are inherited in a Mendelian manner, the dsRNA viruses and dsDNA VLEs show non-Mendelian transmission, so mating between a carrier and a noncarrier leads to most or all progeny being carriers (Bevan and Somers 1969, Somers and Bevan 1969). The extra-chromosomal elements can also be lost more easily than nuclear genes (e.g. high-temperature or cycloheximide treatment often leads to loss of dsRNA viruses; Fink and Styles 1972, Weinstein et al. 1993, Carroll and Wickner 1995, Taylor and Bruenn 2009). Another difference between chromosomal and nonchromosomal forms of killer genes is that the latter show competitive exclusion within cells so that a cell will typically have at most only one type of M satellites (Wickner 1983, Schmitt and Tipper 1992). Note that it is possible to construct yeast strains that have cDNA copies of multiple different viral killer genes integrated into their chromosomes in addition to having a viral killer system (Schmitt and Breinig 2006).

As well as providing an important model system for studying yeast molecular and cellular biology, killer viruses and/or their toxins also feature in experimental evolution studies of nontransitive and positive frequency-dependent fitness interactions (Greig and Travisano 2008, Buskirk et al. 2020, Giometto et al. 2021), and killer yeasts are being investigated for their potential applied use for the biocontrol of pre- and postharvest crop losses (Díaz et al. 2020, Hernandez-Montiel et al. 2021), for food preservation (Bedir and Kuleaşan 2021), in the wine industry (Mannazzu et al. 2019), and for the control of human infections (Díaz et al. 2020, Fredericks et al. 2021b, Giovati et al., 2021).

Despite considerable understanding of the molecular mechanisms of toxin production and killing in at least some cases, the adaptive significance of toxin production and killing is unclear (Boynton 2019). For the chromosomal genes that are inherited in a Mendelian manner, whether they spread or not will depend on their effect on the fitness of the yeast cell (e.g. fitness costs of toxin production and fitness benefits of reduced competition for resources from those cells killed). We can therefore expect that chromosomally integrated genes encoding killer function have the effect of increasing the average fitness of the cells carrying them. However, for the dsRNA- and dsDNA-encoded toxins there are additional or alternative considerations. They can increase in frequency due to drive (non-Mendelian inheritance), rather than due to increasing host fitness. Moreover, cells with these extra-chromosomal elements will occasionally give rise to mitotic or meiotic daughter cells not containing the element, and the killer toxin may simply function to kill these cells, as a way of maintaining the infection, analogous to the addiction mechanisms of some bacterial plasmids (Kast et al. 2015, Wickner and Edskes 2015). On the parsimony principle of assigning adaptation to the lowest level compatible with the facts (Williams 1966, Williams 1985), while toxin production should be of benefit to the dsRNA satellite encoding it, it may be of no benefit to the host cell, or even the helper virus, and they may instead be parasites.

*Saccharomyces paradoxus* is a well-studied forest yeast typically associated with oak trees (Johnson et al. 2004, Koufopanou et al. 2006, Kowallik et al. 2015, Kowallik and Greig 2016). Unlike *S. cerevisiae*, it has never been domesticated (Liti et al. 2009). The ‘Silwood’ population of *S. paradoxus* in the South-East UK has been particularly intensively studied for genetic diversity, clonality, mating system, small-scale differentiation, and factors affecting rates of molecular evolution (Johnson et al. 2004, Koufopanou et al. 2006, Replansky et al. 2008, Tsai et al. 2008, Koufopanou et al. 2015, Koufopanou et al. 2020). Previous surveys for the killer phenotype in *S. paradoxus* have shown it is present in a minority of strains, including in the Silwood population, and that it is due to M satellites of L-A virus (Pieczynska et al. 2013, Chang et al. 2015, Rodríguez-Cousiño et al. 2017, Boynton et al. 2021). In their study of L-A virus diversity in *S. paradoxus*, Rodríguez-Cousiño et al. (2017) sequenced three M satellites, M62, M21, and M74, in the Silwood population.

In this paper, we report on an expanded survey of the killer phenomenon and dsRNAs in the Silwood population of *S. paradoxus*, to characterize their frequency and diversity at the molecular level. We identified multiple killer and nonkiller strains harbouring dsRNAs, confirmed the causal role of M dsRNA in killing activity through cycloheximide-mediated curing, and pool-sequenced dsRNAs from 17 killer and nonkiller strains. Six different M satellites (three previously described and three new ones) were found, with no detectable sequence homology to each other, all with the expected RNA sequence motifs and with ORFs. The amino acid sequences of the new M satellites, however, were variable in the presence or absence of structural features previously found in the preprotoxins of killer toxins. We also assembled eight L-A viral sequences, all of which encoded proteins with the expected conserved domains and motifs. For both M and L-A dsRNAs, we were able to assemble 5′ upstream regions extending further than in previous studies, revealing conserved patterns of direct and inverted repeats and insertion–deletion polymorphisms. These data provide a detailed description of the molecular diversity of dsRNA viruses and their satellites in a natural yeast system, as a first step towards further understanding of the evolutionary and ecological forces acting upon them.

## Materials and methods

### Strains and media

Sixty strains from our culture collection were used in this study (Appendix 1). They were collected in 1996–2004 from a small area straddling the Silwood Park campus of Imperial College and neighbouring Windsor Great Park, with a maximum distance between collection sites of 3.5 km (Johnson et al. 2004, Koufopanou et al. 2006). To assay the killer phenotype, we used as a tester the *S. cerevisiae* strain M984 *(MATa/MATα lys11/+ +/ilv3 +/leu2 [KIL-0*]) (Tallóczy et al. 1998), which is sensitive to all killer strains in *S. cerevisiae* (kindly provided by Duncan Greig). Yeasts were grown in YEPD medium (1% yeast extract, 2% peptone, and 2% dextrose), either liquid or solid (with 1.8% agar).

### Killer assay

The killer assay was performed on methylene blue (MB) agar (YEPD supplemented with 1.407% citric acid, 1.896% K₂HPO₄, and 0.003% methylene blue) at three different pH values (4.2, 4.5, and 4.7). To assay killer and nonkiller states, 5 μl of each yeast strain grown overnight in YEPD was spotted onto MB agar plates. The known sensitive strain (*S. cerevisiae* M984), grown overnight in the same medium, was diluted 1:10 in YEPD, transferred to a sterilized spray bottle, and sprayed onto the plates either on the same day that the tester strain was spotted (same-day assay) or 2 days later (2-day assay). After spraying, plates were incubated at 22°C for 48 h. Killer activity was detected by inhibition of growth of the sensitive strain around the tester colony, a reduction in colony number or a colour change to blue. Strains displaying a killer phenotype in three or more tests were classified as killers, whereas strains showing the phenotype fewer times were retested to confirm or exclude the killer phenotype. The use of both assay timings and multiple pH conditions increased the range of environmental conditions under which the killer phenotype was tested, to reduce the likelihood that a killer phenotype would be missed.

### Cycloheximide treatment

To cure strains of the M dsRNA, they were grown on YEPD medium containing 2 μg/ml of cycloheximide and incubated at 30°C until colonies appeared. Three clones from each strain were then cultured in YEPD broth overnight and their killer activity was tested using the MB agar diffusion assay. All strains were assayed on both same-day and 2-day assays and the dsRNA of one of the nonkiller clones from each strain was extracted and analysed by agarose gel electrophoresis.

### dsRNA purification and visualization

dsRNA was extracted using the RiboPure™ Yeast Kit (Amicon). The amount of sample used per extraction was increased to five times that recommended in the kit instructions, and the amount of elution buffer used to elute the RNA from the column was doubled. To remove ssRNA, the extracted dsRNA was treated with lithium chloride as follows. An equal volume of 4 M lithium chloride (Sigma) was added to the extracted dsRNA, and the samples were incubated at 4°C overnight. After centrifugation at 16 000 × *g* for 30 min, the supernatant was transferred to a new tube, and an equal volume of isopropanol and one-quarter volume of 7.5 M ammonium acetate were added. The samples were then incubated at −20°C for 2 h, centrifuged at 16 000 × *g* for 10 min*,* and the pellets were washed with 70% ethanol. After drying, the pellets were dissolved in RNase-free water. The extracted dsRNAs were then treated with DNase I (Sigma) to remove DNA contamination; this DNase has 6% RNase activity, which would have removed any remaining ssRNA. The enzyme was then removed using the RNeasy Kit (Qiagen). The dsRNA was visualized on a 1% agarose gel using GelRed (Sigma) and the concentration was quantified using a NanoDrop.

### S1 nuclease treatment

S1 nuclease was initially used to remove single-stranded RNAs (ssRNAs). However, we observed that at high temperatures, S1 nuclease can cleave most of the Ms in the middle. As a result, to prevent potential damage to dsRNA, S1 nuclease was not used for ssRNA removal. Instead, it was used to determine the differences between M dsRNAs. The purified dsRNA was incubated for half an hour with 1 unit of S1 nuclease (Promega) per μg of nucleic acid at 67°C. The products were then visualized on an agarose gel, as described above.

### cDNA preparation and sequencing

Two libraries were prepared for sequencing of dsRNA viruses, one from strain Q62.5 alone and one from a pool of 17 strains carrying dsRNAs, including Q62.5 as a control for the bioinformatics analysis. The dsRNA from Q62.5 was treated first with DNase I and then the cDNA was prepared by GATC Biotech. The dsRNAs for the pool were first treated with DNase I followed by TURBO DNase (Ambion) to remove any remaining DNA contamination. After removal of the enzymes using the RNeasy Kit (Qiagen), the cDNA for the pool was prepared in our lab as follows. Based on the results from the NanoDrop assay and the intensity of the bands on the gel, the amount of dsRNA from each strain was adjusted to equalize concentrations across the pool. The first strand was synthesized using the SuperScript III First-Strand Synthesis System (Invitrogen), following the manufacturer’s instructions except that after the dsRNAs from the 17 strains were mixed with random hexamer primers and dNTPs, the mixture was denatured at 99°C for 5 min and snap frozen in a −80°C ethanol bath. The second strand was then synthesized using the NEBNext® mRNA Second Strand Synthesis Module (BioLabs). The cDNA was then purified using the QIAquick PCR Purification Kit (Qiagen), visualized on a 3% agarose gel, and the concentration was measured using the Qubit® dsDNA HS Assay Kit. Both libraries were then sequenced by GATC Biotech using the Illumina MiSeq platform.

### Bioinformatic analyses

The sequence data were analysed in Geneious v9.1 (www.geneious.com) using both the Geneious de novo assembler and the Tadpole assembler (version 35.82) implemented in Geneious, and the results were compared between the two approaches. Sequences from the Q62.5 library were assembled using the normal settings of Geneious. Since the pooled library had a mixture of dsRNAs from different strains and the identity of the different L-A variants was high, metagenomic settings for mapping to a reference and *de novo* assemblies were optimized based on the L and M in Q62.5, which was included as a control in the pool. The dsRNAs were assembled using the *de novo* assembly settings and tested or extended using optimized ‘mapping to a reference’ settings. In general, relaxed mapping parameters were applied to detect variation within individual strains, whereas stricter settings were used to ensure the reliability and accuracy of the final sequences. The M satellites have a long tract of poly(A), which interrupted the assemblies, meaning that we could assemble fragments before and after the poly(A), but not make the pairs.

The ORFs of dsRNAs were initially identified in Geneious using the standard codon settings, which include UUG and CUG as potential initiation codons. Subsequently, we screened for alternative yeast initiation codons as described by Hernández et al. (2019).

BLAST, BLASTp, tBLASTn, and PSI-BLAST searches were performed against the NCBI nonredundant (nr) database using default parameters.

**Figure 2:**
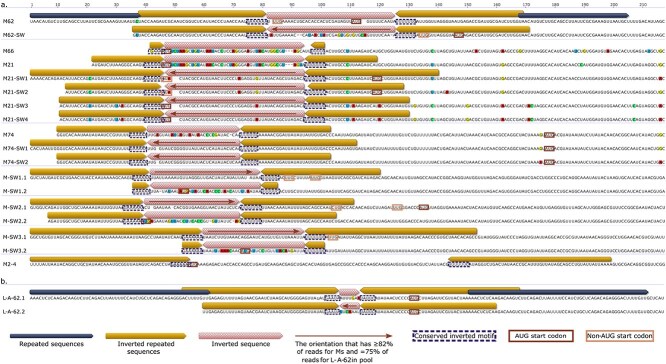
5′ end structural features of (a) different Ms in this study; M2–4 from *S. cerevisiae* (Ramírez et al. 2017) and the M66 virus from Lithuania (Vepštaitė-Monstavičė et al. 2018) are also included for comparison and (b) two L-A-62 variants. M62 and L-A-62.1 are from the Q62.5 library, and the rest of the sequences are from the sequencing pool. Alignments for each type of M are shown separately and highlights show differences from the consensus for each type. The upstream sequence of the inverted repeat in M2–4 was removed to improve the presentation of the sequence in the figure. The inverted repeats flanking the inversion polymorphism, also extend to varying degrees into the pink inverted regions (between the conserved inverted motifs).

**Figure 3:**
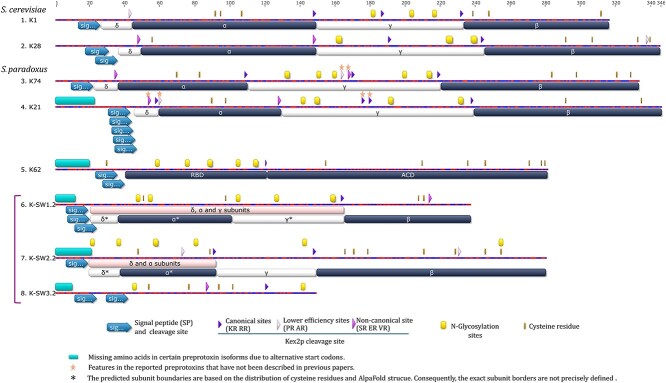
Post-translational modification features of previously characterized preprotoxins K1 (*S. cerevisiae*), K28, K74, M21, and M62 (*S. paradoxus*) (Riffer et al. 2002, Gier et al. 2020, Rodriguez-Cousiño et al. 2022) and predicted features of proteins encoded by M satellites newly identified in this study (purple bracket). Only the longest isoform of each preprotoxin is shown. The putative receptor-binding domain (RBD) and aerolysin core domain (ACD) have been suggested for K62. It remains unclear whether the Kex2p/Kex1p site between them is active (Creagh et al. 2025a).

Figures 1–3, 5, and 6, as well as several supplementary figures, were generated in Geneious v9.1 (www.geneious.com).

### Predicting functional sites in the preprotoxins

The various sites needed for processing of preprotoxins were detected manually and using Geneious, as follows. The signal cleavage sites were detected using the prediction tool in Geneious and the signal peptide that had the highest score was selected as the cleavage site. N-glycosylation sites, cysteine residues, and Kex2p/Kex1p sites were searched for manually. N-glycosylation occurs at N-X-S/T motifs and Kex2p cleavage usually occurs most efficiently after Lys-Arg (K-R) and Arg-Arg (R-R) and with much lower efficiency after Pro-Arg (P-R) and Ala-Arg (A-R) (Brenner and Fuller 1992). However, it was shown in *S. cerevisiae* that cleavage after Ser-Arg (S-R) and Glu-Arg (E-R) in the K28 is as efficient as cleavage after Lys-Arg (K-R) (Riffer et al. 2002). In M74 from *S. paradoxus,* Val-Arg (V-R) was detected as a noncanonical Kex2p/Kex1p recognition site (Rodriguez-Cousiño et al. 2022). In our figures, these cleavage sites are called ‘canonical site’, ‘lower-efficiency site’, and ‘noncanonical site’, respectively.

After removal of the signal peptide, the tertiary structures of the predicted proteins encoded by the identified M satellites were predicted using AlphaFold 3 (https://alphafoldserver.com). The resulting models were then searched against the DALI (http://ekhidna2.biocenter.helsinki.fi/dali/) and Foldseek (https://search.foldseek.com/search) servers to identify structural homologues.

In the new preprotoxins, where the number of detected Kex2p/Kex1p sites was insufficient for predicting the subunits, the approximate cleavage sites between subunits were predicted based on the cysteine residues (which are found in the α and β subunits in all three studied M satellites) and the tertiary structure of the preprotoxin.

### Nomenclature

There are three types of Ms in the pool that have previously been reported (Rodríguez-Cousiño et al. 2017): M62, M21, and M74. Sequences identical to the reported sequences were named accordingly, and their variants were named using the type name followed by SW (for Silwood Park—Windsor Park). Depending on how many variant sequences have been found, they are given a number after that. For example, there were five variant sequences of M21 in the pool and they are called M21, M21-SW1, M21-SW2, M21-SW3, and M21-SW4. There were also new types of M found in the pool and, as it is not possible to assemble sequences across the poly(A), we treat sequences before and after the poly(A) separately. Three new types of M based on sequences before the poly(A) were found in the pool. Their names start with M followed by SW (for Silwood–Windsor), followed by a number for the type and a number for the variant sequences: M-SW1.1 and M-SW1.2, M-SW2.1 and M-SW2.2, and M-SW3.1 and M-SW3.2. The types are largely nonalignable with each other, whereas different variants of the same type are easily alignable. We propose these as names for the M satellites. In addition, two new sequences after the poly(A) were assembled, which we call APA1 and APA2 (for After Poly A). These are not names of viruses but simply names for sequences. For the L-A viruses, we assembled five novel variants that we call L-A-SW1, L-A-SW3, L-A-SW4, L-A-SW5, and L-A-SW6. Apart from L-A-SW4, all L-As in the pool had two variant sequences with different 7-nt motifs at the 5′ end, AUUUGAA and UUCAAAU, and the former were given .1 at the end of their names and the latter .2.

## Results

### Phenotypic survey

Sixty Silwood and Windsor strains of *S. paradoxus* were tested for their effect on a known sensitive strain of *S. cerevisiae*. The tests were done by spotting the strain to be tested on a plate and then spraying on the sensitive strain either on the same day or 2 days later and then waiting 2 days. The assays were done at three different pH values (4.2, 4.5, and 4.7). Of the 60 strains tested, 16 (27%) showed some killer activity in one or both of the two assays (Appendix 1), with apparently considerable variation in the strength of the killer activity among strains (Supplementary Fig. S1). Eighteen of the strains had been tested previously for killer activity (Pieczynska et al. 2013, Chang et al. 2015), and for 17 strains, there was perfect correspondence with the previous results (4 killers and 13 nonkillers). For one strain (Q59.1), no killer activity had previously been detected (Pieczynska et al. 2013, Chang et al. 2015), whereas we did observe it. The nuclear genomes of a number of these yeast strains have previously been sequenced (Koufopanou et al. 2020), including three pairs of strains that were essentially identical and likely to be clonemates, and all three pairs were concordant (1 pair killer; 2 pairs nonkiller; Appendix 1).

### dsRNA virus survey

Twelve of the 16 killer strains and 18 of the 44 nonkillers were then screened for the presence of dsRNAs, and 17 strains were positive, all 12 of the killer strains and 5 of the 18 nonkiller strains (Appendix 1 and Supplementary Fig. S2). Each strain with dsRNA viruses showed two bands. Based on the DNA ladder, the length of the larger band (the putative L-A virus) was ~4500 bp, while the other (the putative M satellite) showed considerable variation in length among strains, ranging from 1000 to 2500 bp (Supplementary Fig. S2). The two shortest Ms were both from nonkiller strains, Q95.3 and Q16.1, with sizes around 1–1.4 kb.

Further variation in the M dsRNA was observed in their response to S1 nuclease digestion (Appendix 1 and Supplementary Fig. S3). S1 nuclease cuts single-stranded RNAs, and the most likely place to cut in these dsRNAs at high temperature is the poly(A), which should have a lower melting temperature than other regions (Welsh and Leibowitz 1982). The M dsRNA was completely cleaved into two fragments in all strains tested, except for three of the five nonkiller strains: Y2.8 (no digestion), and Q95.3 and Y1 (both showing partial digestion). These differences may reflect a smaller or absent poly(A). Thus, based on length and response to S1 nuclease, it was clear that there were a number of different types of M in the population, among both killer and nonkiller strains.

### Curing the M satellite

To test whether the M dsRNA is responsible for the killing activity, we grew the 12 killer strains on YPD plates containing 2 μg/ml cycloheximide, which can cause yeast cells to lose their M dsRNA (Fink and Styles 1972, Carroll and Wickner 1995). Three clones from each strain were then tested for killer activity in both same-day and 2-day assays. In the vast majority of cases, no killer activity was seen and, for all strains, at least one clone showed no killer activity in both tests. We then extracted dsRNA from these nonkiller strains, and in all cases, the L-A virus was still present but the M had disappeared (Appendix 1 and Supplementary Fig. S4).

### dsRNA sequencing: Ms

#### Overview

As an initial test, we sequenced the dsRNA from a single strain, Q62.5, and produced a *de novo* assembly of the M dsRNA. Our initial analyses focused on the region starting from the well-conserved GA_6_ motif (i.e. a G followed by 6 As) that is thought to be used in transcription initiation (Rodríguez-Cousiño et al. 2011). The sequence was identical to that previously published (Rodríguez-Cousiño et al. 2017), and no SNPs were found among our reads with frequency > 1%. We then sequenced dsRNAs from a pool of all 17 strains, including Q62.5 (as a control), two other strains with viruses that have previously been sequenced (T21.4 and Q74.4), nine other killer strains and five strains with no detectable killer activity that carried dsRNA viruses. We performed *de novo* and mapping to reference assemblies using 27.6 million reads. As it was not possible to assemble across the poly(A) sequence in the middle of the Ms, we have separate assemblies for sequences before the poly(A) (where any gene coding for a toxin would be located) and those after. Among the former, six different dsRNAs were identified, including exact matches to the already published M62, M21, and M74 dsRNAs, validating the assembly process, and three completely new Ms, all encoding proteins with no detectable sequence-level homology to each other (Table 1).

**Table 1:** Overview of M satellite sequences found in the pool (there are 17 strains in the pool. See Appendix 1).

Satellite	Length (from GA5–6 to poly(A); nt)	Inferred preprotoxin length (aa)	Variants	Effect variant on amino acid sequence
M62	986	261–272	2 sequences; identical except for a 41 bp inversion that results in differences at 25 sites after the GA_6_	Change in position of start codon
M21	1 154–1175	323–346	5 sequences: pairwise differences at 2–131 sites (0.2%–11.2% divergence). Starting sequences fall into 2 types differing due to a 45 bp inversion, resulting in 20 nucleotide differences between M21 and the new variants after GA_6_.	Change in position of start codon; scattered amino acid differences elsewhere
M74	1 271	333	3 sequences: 2 of the 3 sequences identical except for a 31 bp inversion resulting in differences at 10 sites after the GA5; the third sequence is identical to one of them in this region but differs from both other sequences at 11 sites elsewhere in the sequence.	No change in start codon; 5 amino acid differences
M-SW1	1 093	222–237	2 sequences: Identical except for a 37 bp inversion that results in differences at 12 sites after the GA_5_.	Change in position of start codon
M-SW2	956	249–280	2 sequences: Identical except for a 31 bp inversion that results in differences at 17 sites after the GA_6_.	Change in position of start codon
M-SW3	716	139–149	2 sequences: Identical except for a 35 bp inversion that results in differences at 13 sites after the GA_6_	Change in position of start codon

All three new Ms had GA_5_ or GA_6_ near the 5′ end, a coding sequence, and a poly(A), and appeared in the output from both the Geneious and Tadpole assemblers. We call these new dsRNAs M-SW1, M-SW2, and M-SW3. Two of the novel Ms are of comparable length to the previously published ones, but one, M-SW3, is significantly shorter than the others, both in nucleotide length from GA_5_ to poly(A) and in the length of the ORF (Table 1).

#### Nucleic acid structural features and polymorphisms

In all Ms, the GA5 or GA6 motif is repeated downstream in the reverse form, i.e. U_5_C or U_6_C. We also found this conserved inverted motif structure in all M satellites of *S. cerevisiae* (M2–4 shown in Fig. 2 as an example). Moreover, for all our Ms, we found sequence reads that differed in the orientation of the intervening sequence between the inverted motifs, usually at markedly different frequencies (Fig. 2). A minority of reads (4%–12%) in the assemblies showed the orientation previously reported for the published M dsRNA. This pattern was also observed in the new Ms (one orientation had a higher frequency than the other).

Two types of M showed single-nucleotide variation in addition to the inversion polymorphism. M21 showed the highest diversity (5 variant sequences), followed by M74 (3 variant sequences). The variant sequences of M21 are shown in Supplementary Fig. S5, and the nucleic acid and amino acid distance matrices for the ORFs in Supplementary Fig. S6a. We also included in the alignment the published sequence of the M66 satellite from a *S. paradoxus* strain from a natural fermentation of serviceberries in Lithuania (Vepštaitė-Monstavičė et al. 2018), which is very similar to M21 (91% ORF nucleotide identity). The sequence between the inverted motifs of M66 and M21 differed at three nucleotide positions. Interestingly, the reverse orientation of the M66 sequence was found in M21-SW3 and M21-SW4 from the UK. Between the GA_6_ and the poly(A), 220 of 1179 nucleotide sites are variable, and 50 of 347 amino acid sites. Eleven of these are in the first 16 amino acid positions, due to the inversion encompassing the beginning of the coding region. Phylogenetic analysis shows that the Lithuanian serviceberry sequence is the most divergent (Supplementary Fig. S6b); otherwise, the tree divided the variant M21s into a group of two closely related sequences (M21-SW3 and M21-SW4, differing at two nucleotide positions) and a group of three more distantly related sequences (M21, M21-SW1, and M21-SW2, differing at 12–44 positions; differences between groups occurred at 85–114 positions).

M74 and M74-SW1 are identical except for the orientation of the inversion, resulting in 10 nucleotide differences, while M74-SW2 differs from the other two sequences at 11 sites elsewhere in the genome, resulting in five amino acid differences (Supplementary Figs S7 and S8). The rest of the Ms had two variants each, differing by the inversion, leading to variable sites in 31–45 nucleotides (see Table 1 and Supplementary Figs S9–S12).

The inversion between the GA_5–6_ and U_5–6_C motifs can also affect the position of start codons. Typically in Ms, the killer toxin ORF starts with an AUG codon close to the conserved GA_5–6_ (SCHMITT and TIPPER 1995). All our Ms follow this pattern except M74, which starts at nucleotide 142 (Rodriguez-Cousiño et al. 2022). In the remaining Ms, the inversion affects the position of AUG and/or non-AUG start codons (Fig. 2).

#### Preprotoxin structural features and effects of variable sites

**Figure 4:**
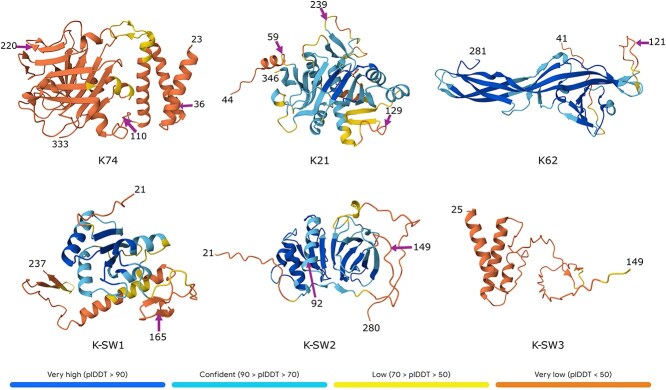
AlphaFold-predicted tertiary structures of proteins encoded by the studied Ms following removal of the pre-region. Structures are coloured by pLDDT confidence scores (the scale is shown below the figure). Arrows indicate the positions and residue numbers of predicted Kex2p/Kex1p cleavage sites between putative subunits. Residue numbers for the N- and C-termini are also indicated. Notably, the structure of M74 was previously reported to also exhibit relatively low confidence (Creagh et al. 2025a).

The ORFs of each M satellite and its variants were translated *in silico*, and the predicted preprotoxin sequences were analysed for motifs known to be involved in the post-translational processing of the previously described killer toxins K1 and K28 (*S. cerevisiae*) and K74, K21, and K62 (*S. paradoxus*) (Fig. 3; Supplementary Figs S5, S7, and S9–S12) (Riffer et al. 2002, Gier et al. 2020, Rodriguez-Cousiño et al. 2022, Creagh et al. 2025a, Creagh et al. 2025). In addition, their 3D structures were predicted using AlphaFold following removal of the signal peptide (pre-region), and the resulting models were examined for their overall structural organization (Fig. 4).

For those already sequenced (K62, K21, and K74) our predicted structural motifs were consistent with published predictions (Creagh et al. 2025a, Creagh et al. 2025). In K74, we also predicted a signal peptide at the N-terminal sequence (when the minimum threshold score was reduced from 3.5 to 1.3) and therefore could predict the cleavage site between the pre-region and δ subunit (Fig. 3).

The predicted structural features of the amino acid sequences of the three newly identified proteins, K-SW1, K-SW2, and K-SW3 are shown in Fig. 3. For K-SW1 and K-SW2, we were able to predict some of the preprotoxin subunits, though with less confidence as not all the Kex2p/Kex1p sites were present. Additional noncanonical Kex2p/Kex1p cleavage sites may be present, similar to those reported for K28 and K74, or alternatively, these proteins may have different structural arrangements as proposed for K62. It is also possible some cleavage sites have been lost due to mutation or deletion. The predicted N-glycosylation sites of the K-SW1 and K-SW2 proteins are distributed across the γ and other subunits, unlike those of previously described killer toxins, which are typically restricted to the γ subunit (Riffer et al. 2002, Gier et al. 2020). K-SW1 contains two Kex2p/Kex1p cleavage sites, one of which is a noncanonical site located after the cysteine residues on the predicted β subunit. As a result, only the canonical site, Arg165, was considered as a cleavage site (between γ and β) in this protein. In K-SW2, there are four Kex2p/Kex1p cleavage sites, two of which are lower-efficiency sites and located between cysteine residues that are likely associated with the α and β subunits. The other two, Arg92 and Arg149, are therefore considered as cleavage sites between the α, γ, and β subunits. No subunit was predicted for K-SW3 as none of its features followed the pattern of the known preprotoxins, and its length is about half of the known ones (Fig. 3 and Figs S10–S12).

Comparison of the AlphaFold predicted tertiary structures of the preprotoxins shows that K-SW1 and K-SW2 have overall high-confidence structural models, particularly in the α region of K-SW1 and the α and β regions of K-SW2 (pLDDT > 70–90) (Fig. 4). The structure of K-SW1 and K-SW2 seems closer to K74 and K21 than to K62. In contrast to K62, which shows a more extended conformation, K-SW1 and K-SW2 show intrinsically disordered segments distributed around a more compact structural core. Similar to other preprotoxins, the predicted Kex2p/Kex1p cleavage sites thought to separate subunits of the K-SW1 and K-SW2 proteins are located in intrinsically disordered regions and appear to be surface-exposed and structurally accessible. The predicted α and β regions are well structured, whereas δ and γ regions appear more intrinsically disordered. The first 12 amino acids at the N-terminus of K-SW1 and the first 18 amino acids of K-SW2 show low structural confidence and appear not to form a stable secondary structure. These regions likely correspond to part of a δ subunit. In K-SW1, the region corresponding to the α subunit shows a more well-defined structure and of higher confidence than the β region. In K-SW2, most of the α and β regions display well-defined structures with high confidence, except for the final 39 amino acids at the C-terminus of the β subunit. The AlphaFold model of K-SW3 gives predominantly low confidence scores (pLDDT < 50), indicating the predicted structure is largely unreliable and suggestive of intrinsic disorder. The model lacks a compact globular core and consists mainly of intrinsically disordered, poorly defined secondary structure elements.

The amino acid alignment of predicted preprotoxins from variants of each M shows that, although some of the predicted isoforms have shorter N-terminal sequences (as a result of alternative start codons), they all have all annotations to go through post-translational modification, except K-SW2.1 (Fig. 3 and Supplementary Fig. S11). All isoforms of this preprotoxin are missing the only signal peptide that exists close to the N-terminal sequence.

Apart from the K74 and K21 preprotoxins, there were no amino acid differences among variants of all other types. All functional sites are conserved among variants of K74 except for the N-terminal signal peptide (Supplementary Fig. S7). This putative signal peptide is missing in the K74-SW2 variant sequence due to an amino acid change (A → T) at position 14 of the sequence. This variant would likely be unable to enter the secretory pathway and pass through the ER.

K21 variants show the following changes in functional annotations (Supplementary Fig. S5): (a) two amino acid differences at residues 24 (M → I) and 28 (F → L) in K21-SW3 and K21-SW4, which created four additional signal peptide cleavage sites in these two variants; (b) an amino acid change at residue 128 (E → Q) in the Kex2p/Kex1p cleavage site, between α and γ subunits, in M66 that may reduce its killer activity (or may indicate that a noncanonical site is used, as the carrier strain is found to be killer (Vepštaitė-Monstavičė et al. 2018)); and (c) an amino acid change (N → D) in residue 191, affecting the third N-glycosylation site of K21-SW3 and K21-SW4, may eliminate glycosylation at this position, and therefore may affect the killer and/or immunity phenotypes.

#### Sequence and structural homology analysis of the novel M satellites

Nucleotide and amino acid sequences of the newly identified M satellites were initially analysed using BLAST, BLASTp, tBLASTn, and PSI-BLAST against the nonredundant database. None of the searches revealed any homology to known killer toxin families. Weak similarities to uncharacterized proteins from yeasts and other fungi were identified for both K-SW1 and K-SW2 in tBLASTn and PSI-BLAST analyses. In addition, tBLASTn detected 34% identity to several chromosomal regions in *S. cerevisiae* for K-SW2. No homologous sequences were detected for K-SW3.

The AlphaFold-predicted structures of the predicted preprotoxins were analysed using the DALI and Foldseek servers to identify proteins with potential structural homology. Among them, only K-SW2 showed structural similarity to known yeast killer toxins. In DALI, it exhibited a limited structural resemblance to the *Williopsis mrakii* killer toxin (Z = 3.4; RMSD = 2.5 Å over 71 residues; ~14% sequence identity), suggesting limited similarity at the fold level. However, the low Z-score does not support a confident inference of structural homology. Foldseek also retrieved two known killer toxins (Killer toxin HM-1 from *Cyberlindnera mrakii* and a killer toxin from *C. saturnus*) among the matches; however, both hits showed very low probability values (0.47 and 0.41) and high E-values (3.97e-1 and 3.13e-1), indicating that the similarity is not strong enough to support confident structural homology. Taken together, the DALI and Foldseek results provide no strong structural evidence that the putative preprotoxin is homologous to known killer toxins. The top hits in Foldseek for K-SW1 and K-SW2 with probabilities above 0.97 also corresponded to uncharacterized proteins in yeast species. No significant hits were detected for K-SW3 in either DALI or Foldseek analyses.

#### Sequences after the poly(A)

The non-coding sequences downstream of the poly(A) tracts could not be assembled with the sequences before the poly(A) tracts and therefore needed to be analysed separately. For two of the previously published sequences (M62 and M74), we recovered the expected sequence from the *de novo* assemblies and/or by mapping to reference. Two variant sequences of M74, M74-APA1 and M74-APA2, were also found (Supplementary Fig. S13). For M21, the published sequence has a 371 bp noncoding region downstream of the poly(A), but we were unable to assemble an identical sequence even by mapping to reference. The *de novo* assemblies also produced two sequences that appear to be from M satellites after the poly(A): the sequences started with poly(A), did not have an ORF, and their length was comparable to other post-poly(A) sequences. Both sequences were assembled by both Geneious and Tadpole. Presumably, they are from the newly described Ms (M-SW1, M-SW2, or M-SW3), but we cannot determine which ones. We called these sequences APA1 (1064 bp) and APA2 (955 bp) (Supplementary Fig. S14).

### dsRNA sequencing: L-As

As with the Ms, we first assembled an L-A sequence from the single strain Q62.5, and this was identical to the previously published one (Rodríguez-Cousiño et al. 2017), with no SNPs found among our reads with a frequency > 1%. For the pooled sequence dataset, we initially focused our analyses starting from a GAAUA motif that is well conserved in our L-As from *S. paradoxus* and presumably has a similar function (i.e. possibly acting as a transcription initiation signal) to the GA_5–6_ of Ms and of L-As from *S. cerevisiae* (Rodríguez-Cousiño et al. 2011). Again, the inverse complement sequence (UAUUC) is found downstream, though the separation between the pair of conserved inverted repeats was much smaller compared to the Ms (7 bp versus 31–42 bp) (Fig. 2), and the sequence was in two orientations (AUUUGAA and UUCAAAU) in the assembly of all L-As except L-A-SW4 (which only had reads with the UUCAAAU orientation). Similar to Ms, one orientation is more frequent than the other, averaging around 75%; however, the less frequent orientation occurs at a higher frequency than in Ms. This variation in orientation may result from *in vivo* replication by RNA-dependent RNA polymerase (RdRp) or may arise as an artifact of cDNA preparation.

*De novo* assemblies from the pool combined with mapping to reference recovered the published L-A sequences from Q62.5, T21.4, and Q74.4 (called L-A-62, L-A-21, and L-A-74, respectively (Rodríguez-Cousiño et al. 2017)). In addition to the published L-A-21 sequence, we also found a variant differing at two nucleotides (L-A-SW7). For L-A-74, we also found a sequence identical to the published one, plus another (L-A-SW2) that had one difference from the published sequence and 12 ambiguous nucleotides, which may indicate that there are two sequences that differed at those 12 bases (Supplementary Fig. S15). In addition to the three published sequences (and slight variants thereof), we also recovered five more divergent L-A sequences. These are all alignable and show 2.9% to 18.4% nucleotide divergence across their ORFs. We called them L-A-SW1, L-A-SW3, L-A-SW4, L-A-SW5, and L-A-SW6. No L-BC elements were detected in this population, although L-A and L-BC are known to co-exist in *S. cerevisiae* (Rodríguez-Cousiño and Esteban 2017, Ramírez et al. 2022).

The L-A ORFs from the UK were aligned with 25 previously published L-A sequences from around the world (including L-A-62, L-A-21, and L-A-74; Supplementary Table S1 and Supplementary Fig. S15) using MUSCLE. All sequences from the UK are similar to each other, with >96% identity at the amino acid level (Supplementary Fig. S16), and both the frameshifting and packaging domains are well conserved in the nucleotide alignment (Supplementary Figs S17 and S18). At the amino acid level, all UK sequences have the essential cap-snatching residues (including His154 and four other crucial residues, Tyr150, Asp152, Tyr452, and Tyr538), and all eight conserved motifs in RdRp (Routhier and Bruenn 1998) (Supplementary Fig. S19).

**Figure 5:**
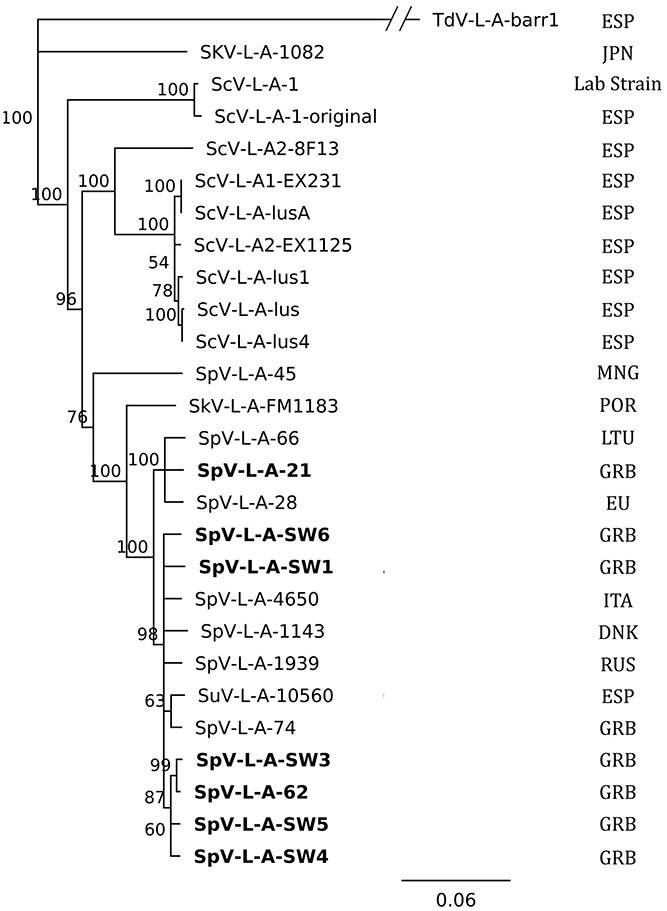
Phylogenetic relationships of L-A viruses from around the world. Neighbour-joining tree of amino acid sequences of the Gag-Pol fusion protein. The sequence of TdV-LAbarr1 from *Torulaspora delbrueckii* was selected as the outgroup (see also Table S1). Strains from the UK are shown in bold; numbers on branches are bootstrap values; the scale bar represents 0.06 substitutions per amino acid site.

Phylogenetic analysis shows the UK sequences forming a clade along with all other L-As from European strains of *S. paradoxus* and an L-A from *S. uvarum* (Fig. 5). Sequences from the Far East lineage of *S. paradoxus* (L-A-45) and from *S. cerevisiae* and *S. kudriavzevii* fall outside this clade. This result is consistent with previous reports (Rodríguez-Cousiño et al. 2017, Ramírez et al. 2020). Within the European *S. paradoxus* clade, sequences fall into two groups, each supported by 98%–100% bootstrap values, with nucleotide sequence identity between members of the same group >90%, and between members of different groups of ~82% (Supplementary Fig. S16). Most of the Silwood Park sequences fall into the larger of the two groups, but one of them (from T21.4) is in the other group, indicating no strict geographical split between the groups. Overall, the high similarity of L-As in Silwood Park contrasts with the high diversity of M satellites.

### dsRNA sequencing: M and L-A upstream sequences

**Figure 6:**
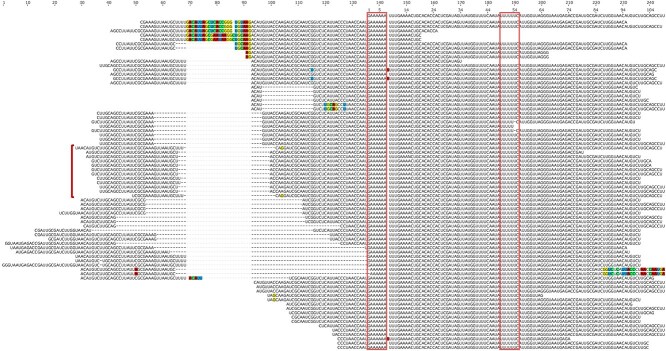
Assembly of Q62.5 library reads at the 5′ end of the M62 sequence, upstream of the conserved GA_6_ motif, showing indel variation. The conserved inverted motifs are boxed and the bracket shows the sequences used in further analyses. The majority of reads are ≥100 bp in length, extending from the 5′ GA_6_ conserved motif and covering 19–153 bp downstream of this motif. These reads are nearly identical to the reference sequence. To allow the alignment to be displayed at a larger scale, 3–53 bp were truncated from the 3′ ends of the 54 reads.

For both M and L-A dsRNAs, we were able to assemble some sequence upstream of their conserved GA_5–6_ or GAAUA motifs, respectively. The length of the extra sequences assembled in the Q62.5 library is greater than those from the pool library, likely due to differences in sequencing methodology and we therefore focus on these. The extra sequences present in both M and L-A dsRNAs required manual realigning of the reads, and in both cases, the alignments revealed abundant insertions and deletions. For M62, only one group of these reads was chosen for further analysis (shown in brackets in Fig. 6). For L-A-62, we were able to assemble a sequence 101 nt upstream of the GAAUA (Fig. 2 and Supplementary Fig. S20). We also recovered a smaller number of heterogeneous reads that aligned with the sequence 75–148 nt downstream of the GAAUA motif. These reads lacked the GAAUA itself and up to 40 base pairs immediately following it. The 5′ ends of these reads, spanning 39–192 nucleotides, showed significant heterogeneity. They could be divided into 12 distinct groups, each showing similarity to sequences from ribosomal RNAs, genomic DNAs, L-A, or M dsRNA (Supplementary Fig. S20).

The 5′ upstream regions of L-A-62 and M62 shared similar structural elements despite having different sequences (Fig. 2). In particular, it turns out that the conserved GA_5–6_ and U_5–6_C motifs in M62 are the inner-most parts of much longer inverted repeats 44 nt in length, and similarly the GAAUA and UAUUC sequences in L-A-62 are the inner-most parts of inverted repeats 55 nt in length (golden bars in Fig. 2). Moreover, these longer inverted repeats are, in turn, flanked by long direct repeats (38 nt in M62 and 62 nt in L-A-62; navy bars in Fig. 2). In both cases, the direct repeats extend to the very end of the assembled sequence, and perhaps *in vivo* extend even further. Extended inverted repeats were also found at the 5′ end of all Ms and L-As in the pool (Fig. 2 and Supplementary Fig. S15). We also identified similar inverted repeats in the previously published M2–4 sequence of *S. cerevisiae*, although they differ by two nucleotides (Ramírez et al. 2017). None of the pool sequences extended far enough upstream to test for flanking direct repeats. Although the sequence upstream of the inverted repeat in *S. cerevisiae* M2–4 is extended, the extended sequence is not matched by an identical sequence downstream.

The inverted repeats also extend to varying degrees into the inverted regions (between the conserved inverted motifs) in Ms, presumably forming a stem-loop structure similar to those previously reported in other M satellites (Thiele et al. 1982, Rodríguez-Cousiño et al. 2011, Rodriguez-Cousiño et al. 2022) (Fig. 2).

## Discussion

Our phenotypic survey of *S. paradoxus* strains from the Windsor–Silwood Park population shows a frequency of 27% killer phenotype among the 60 strains tested, higher than the 5% reported by Boynton et al. (2021) for a German forest population of *S. paradoxus*. Some *S. cerevisiae* populations sampled in vineyards or from collections show much higher frequencies (42%–75%) of killer phenotypes (Hidalgo and Flores 1994, Kapsopoulou et al. 2008, Maqueda et al. 2012, Crabtree et al. 2023). We tested for killer phenotypes in a variety of conditions, but it remains possible that strains for which we did not detect a killer phenotype might show it under some untested conditions.

All our killer strains had dsRNA viruses and curing the satellite of the virus with a cycloheximide treatment removed the killer phenotype, indicating the killer phenotype is due to dsRNA as opposed to some nuclear genes. In addition, 5 of 18 (28%) apparent nonkiller strains also had dsRNA viruses. The overall predicted frequency of strains with dsRNAs is thus 0.27 + (0.73)(0.28) = 47%, and the predicted frequency of dsRNA strains with a killer phenotype is 0.27/0.47 = 57%. For both killers and nonkillers, there were always two bands on the agarose gel, a larger one (L-A) constant in size and a smaller one (M) that was variable in size and variable in response to S1 nuclease treatment. The fact that we did not find Ls without Ms suggests that the Ms either enhance the fitness of the L in this setting or are efficient parasites. By contrast, in a sample of 536 *S. cerevisiae* strains from New Zealand vineyards, 34.6% had Ls without Ms (Travers-Cook et al. 2025); see also Vijayraghavan et al. (2023). To characterize the genomic diversity among viruses, we sequenced the dsRNA from a single strain, Q62.5 and a pool of 17 strains that contained Q62.5 as a control. For M satellites in the pool, we were able to assemble sequences up to the poly(A), and we found six completely different (unalignable) types, three of which were previously sequenced (M62, M21, and M74) (Rodríguez-Cousiño et al. 2017) and three new (M-SW1, M-SW2, and M-SW3). We also assembled four sequences after the poly(A), two previously reported and two new. Future work on individual rather than pooled strains could match these up. In addition, eight L-A variants were found in the pool, all readily alignable to each other and to previously reported L-A sequences with all the previously described key functional domains and motifs well conserved.

In our assemblies, we were able to extend the 5′ ends of both M and L-A dsRNA, particularly in the Q62.5 library, revealing novel structural features. For both L-A-62 and M62, there is an inverted repeat flanked by direct repeats and within the inverted repeats are the well-conserved motifs (GA_5–6_ and corresponding U_5–6_C in Ms and GAAUA and corresponding UAUUC in L-As) (Fig. 2). Our further analysis demonstrated that this conserved inverted motif also exists in all *S. cerevisiae* Ms (one of which, for M2–4, is shown in Fig. 2) and we showed that in M2–4, the conserved sequence is also part of a larger inverted repeat structure. Previous studies have described inverted repeats at the 5′ end of L viruses from *S. cerevisiae* and *T. delbrueckii,* though they differ in their position and spacing (Ramírez et al. 2020). The most similar structure to ours appears in another class of L viruses, the L-BCs of *S. cerevisiae* and *T. delbrueckii*, which have only the inverted repeats (Ramírez et al. 2022). The presence of similar structural features at the 5′ ends of both M and L-A dsRNAs is consistent with their reliance on the same replication and encapsidation system (the Gag and Pol proteins encoded by L-A) and the same host translation machinery (Magliani et al. 1997, Schmitt and Breinig 2006, Schaffrath et al. 2018).

The (−) strand of *S. cerevisiae* L-A dsRNA is thought to contain a *cis*-acting signal within the 25-nucleotide region that begins at the conserved U_5–6_C sequence near the 3′ end and extends toward the 5′ end and is required for transcription of the (+) strand (Fujimura and Wickner 1989). The reverse complement of the conserved sequence is also present in this region and together may form part of this *cis*-acting signal. A similar mechanism likely applies to M satellites, as the same role has been proposed for their conserved U_5–6_C sequence on the (−) strand (Rodríguez-Cousiño et al. 2011, Ramírez et al. 2017). As this region is AU-rich in both L-As and Ms, it probably facilitates the formation of the transcription–initiation complex (Fujimura and Wickner 1989, Rodríguez-Cousiño et al. 2011, Rodríguez-Cousiño et al. 2013).

The inverted repeats in the Ms extend to varying degrees into the region between the two conserved (inverted) motifs and likely form a stem-loop structure similar to those reported in other Ms. On the (+) strand of M1, M-lus, and M74, the sequence from GA_5–6_ to its inverted repeat U_5–6_C can form a stem-loop secondary structure that is energetically stable (Thiele et al. 1982, Rodríguez-Cousiño et al. 2011, Rodriguez-Cousiño et al. 2022). This stem-loop structure has been suggested to play a role in translation initiation in M1 and has been experimentally confirmed to be essential for this process in M74 (Thiele et al. 1982, Rodriguez-Cousiño et al. 2022). As this stem-loop structure is located between the direct and inverted repeat sequences in our *S. paradoxus* L-A and M dsRNAs, it is likely to be part of a larger structure that can be formed by these repeats in both strands of the dsRNAs. This structure may be functionally important not only for transcription and/or translation initiation but also for other functions, such as protecting the 5′ end of the ssRNA from cytoplasmic exonucleases. In M74, which has the longest UTR (142 bp from GA_5_ to the start codon) among all Ms, in addition to the stem-loop structure between the two conserved motifs, three more stem-loop structures can form before the start codon, which are thought to slow down ribosomes passage (Rodriguez-Cousiño et al. 2022). The presence of an inverted repeat in this region could prevent the formation of these inhibitory structures, leading to a conformational change that promotes translation.

Sequences at the 5′ end of both M and L-A dsRNAs showed substantial structural polymorphism in their assemblies, including (a) inversions between the pair of conserved motifs in both libraries, (b) indels upstream of the conserved motifs of M62 in the Q62.5 library, and (c) heterogeneous reads at the 5′ end of L-A-62 in the Q62.5 library showing homology to either host (including rDNA), or L-A, or M sequences (Figs 2 and 6; Supplementary Fig. S20, respectively). Homology to genomic sequences has also been reported in the extended sequences at both ends of L-As and Ms in *S. cerevisiae* (Ramírez et al. 2017, Ramírez et al. 2020).

The main question is whether these observed polymorphisms represent genuine biological variants or are instead artifacts of the sequencing process. One possibility is that the potential secondary structure in this region, including the stem-loop structure between the repeated sequences, may promote template switching during replication by viral RdRp or during cDNA synthesis by reverse transcriptase. The AU-rich sequences surrounding this region may further facilitate this process. Template switching can generate indels and inversions both *in vivo* and *in vitro* (Lai 1992, Nagy and Simon 1997, Simon-Loriere and Holmes 2011). Heterogeneous reads showing homology to the nuclear genome are unlikely to arise *in vivo*, as viral replication occurs within the capsid (Castón et al. 1997), spatially separated from cytoplasmic RNA and the nuclear genome; they are therefore more likely to represent artifacts resulting from ssRNA and DNA contamination during cDNA preparation. Examination of these heterogeneous reads shows that most lack one or both conserved motifs (Supplementary Fig. S20). In contrast, reads with inverted sequences in both dsRNA assemblies and those with indels observed in M62 retain complete conserved motifs. Importantly, these inversions consistently occur at the same position within the conserved motif pairs across all Ms and L-As. This pattern may result from *in vivo* purifying selection acting against variants that have lost their conserved motifs. In any case, the remaining question is why one orientation occurs at a much higher frequency than the other.

If the inversion polymorphism occurs *in vivo*, it would likely affect the expression of the killer phenotype. In most M satellites, the start codons are inside or between the inverted repeats, so changes in orientation result in changes in the location or type of the start codons (Fig. 2) and therefore in preprotoxins of different lengths and perhaps different levels of translation. As observed in other viruses, some M variants have non-AUG start codons upstream of an AUG (Firth and Brierley 2012), which could result in dual-start codon expression, potentially producing preprotoxin isoforms with varying sizes and expression levels. Although some isoforms are shorter, all appear to have all post-translational modification sites required for ER and Golgi processing, except for the putative preprotoxin from M-SW2.1, which lacks a signal peptide in all isoforms.

The identification of three new M satellites expands the known diversity of these elements in *S. cerevisiae and S. paradoxus*. Sequence analyses indicate that these newly identified M satellites are highly divergent from previously described Ms. No significant similarity to known sequences was found in either sequence-based or structure-based analyses. Despite substantial sequence divergence, the proteins encoded by the newly identified Ms have several key features characteristic of previously described preprotoxins that are required for toxin maturation in the secretory pathway, though to varying degrees. A lack of certain canonical Kex2p/Kex1p cleavage sites prevented the confident prediction of all four toxin subunits (Fig. 3). Perhaps noncanonical motifs are present, similar to those reported in K28 and K74 (Riffer et al. 2002; Rodriguez-Cousiño et al. 2022), or different subunit types may be present as suggested for K62 (Creagh et al. 2025). Alternatively, cleavage sites may have been lost through mutation or deletion. Such changes could potentially reduce or eliminate toxin activity. Another notable difference from previously characterized killer toxins is the distribution of predicted N-glycosylation sites. In the known killer toxins, these sites are typically restricted to the γ subunit (Schmitt and Tipper 1995, Riffer et al. 2002, Gier et al. 2020, Rodriguez-Cousiño et al. 2022) but here, they are distributed more widely across the protein subunits. This altered distribution of N-glycosylation sites could affect protein maturation, secretion, or killer and/or immunity functions.

AlphaFold models suggest that K-SW1 and K-SW2 form compact structural cores, with predicted cleavage sites located in intrinsically disordered and surface-exposed regions consistent with accessibility to processing enzymes (Fig. 4). In contrast, the K-SW3 protein appears substantially different. It is considerably shorter than other known preprotoxins and the predicted structure has low confidence. Furthermore, K-SW3 has fewer predicted post-translational modification sites, with a distribution that differs from those of other preprotoxins (Fig. 3). Altogether, these observations suggest the K-SW3 protein may be intrinsically disordered. Consistent with this interpretation, gel electrophoresis showed that the two shortest M dsRNAs were found in two nonkiller strains (Q95.3 and Q16.1), with sizes of ~1–1.4 kb. One possible explanation is that M-SW3 represents a deletion derivative of an ancestral killer M satellite that was not present in our sample. In *S. cerevisiae*, S and NS dsRNA elements are known deletion derivatives of the M1 and M2 satellites, respectively, with S suppressing M1 and NS coexisting with M2 (Somers 1973, Vodkin et al. 1974, Sweeney et al. 1976, Bruenn and Kane 1978, Fried and Fink 1978, Bostian et al. 1980, Cansado et al. 1999).

### Adaptive considerations

Our observations further highlight the mystery concerning the adaptive role of the killer phenotype within yeast populations. The simplest model, or null hypothesis, is that the killer toxin has no significant ecological effect and is just an addiction mechanism, killing cells that did not inherit the virus and satellite, thus facilitating the maintenance of essentially parasitic elements (Kast et al. 2015, Wickner and Edskes 2015). In this case, the only force increasing the frequency of the dsRNAs in the population is their super-Mendelian inheritance or drive through the sexual cycle and is countered by any reduced host replication rate due to toxin production and spontaneous loss. One difficulty with this hypothesis is that previous population genetic work on this same population has shown that the rate of outcrossing is very low (Johnson et al. 2004, Tsai et al. 2008), which implies that drive will be a weak force (Goddard et al. 2001). Conceivably, one possible way to resolve the discrepancy may be that if the population is spatially structured and mating is relatively common among very closely related nearby genotypes, this could be sufficient to maintain a virus and satellite in the population while having a minimal effect on patterns of linkage disequilibrium, making the mating ‘invisible’ from such data. Formal population genetic modelling would be required to test whether such a scenario could be compatible with the data on the frequency of the dsRNA elements and the genomic patterns of linkage disequilibrium. In principle, horizontal (as opposed to vertical) transmission of the dsRNAs among yeast cells could also act to increase their frequency; such transmission can be observed in the lab when there is some loss of integrity of the cell wall (El-Sherbeini and Bostian 1987), and perhaps, this occurs more often in nature than in the lab. With full genome sequencing, it is possible to identify clonemates, and our initial screen included three pairs of previously identified clonemates. These were concordant for killer status, but perhaps a much larger sample would reveal heterogeneity indicative of horizontal transmission.

If the killer phenotype is not (only) an addiction mechanism, then presumably it is providing some other benefit, such as aiding the host cell with competitive interactions (with conspecifics or members of other species), at least in some circumstances. However, this possibility raises its own question: if the killer phenotype is useful for the host cell, why is it not chromosomally encoded? Chromosomally encoded toxins have been described in nature and engineered in the lab (Goto et al. 1990a, Goto et al. 1990b, Goto et al. 1991, Crabtree et al. 2023, Vijayraghavan et al. 2023), and dsRNA-encoded toxins may even have originated from chromosomally encoded genes (Rodríguez-Cousiño et al. 2011, Ramírez et al. 2017, Rodríguez-Cousiño et al. 2017, Fredericks et al. 2021a). If they were chromosomally located, then there would be no competitive exclusion and cells would be able to encode more than one toxin. The dsRNA (as opposed to chromosomal) location of the toxin genes is perhaps the strongest evidence for the hypothesis of addiction mechanism, as opposed to the hypothesis of aid to competition—though even then, it leaves open the question of why the toxin is encoded by a satellite dsRNA as opposed to the main viral genome.

If our understanding of the forces responsible for the persistence of even a single cytoplasmically encoded dsRNA within a population is rudimentary, the discovery of at least six different satellite types in the same population compounds the mystery. It implies there is not a single ‘best’ type (however ‘best’ might be judged) that can go to fixation and instead suggests selection has a frequency-dependent component. In principle, multiple factors could be playing a role in maintaining this diversity:

(1) Satellite–satellite interactions within cells. Whether they function primarily as an addiction mechanism, in intraspecific competition, or in interspecific competition, if cells with different killer types are able to mate with each other, then it will be important whether one typically ‘wins’ and is found in a preponderance of the diploid progeny, and whether such differences are transitive or intransitive. Diversity would be particularly enhanced if there are nontransitive outcomes (as with intransitivity in more conventional competitive interactions—Kerr et al. 2002).
(2) Satellite–satellite interactions between cells. When cells bearing different satellites encounter one another, again, does one typically ‘win’ over the other, and are differences transitive or intransitive? Selection in favour of killer and immune phenotypes may be a complex function of their frequencies—the more frequent a killer phenotype is, the more likely a neighbouring cell also has it, and therefore is not killed, while at the same time the more important it is to be immune to the toxin.
(3) Abiotic adaptations. Killer toxins often work only in a narrower abiotic range (temperature, pH, salt, nutrients, alcohol, etc.) than the host can live in, and perhaps the *S. paradoxus* in Silwood Park exist in sufficiently diverse abiotic micro-environments that different killer toxins are selected for accordingly (even if it is just an addiction mechanism).
(4) Biotic adaptations. If the killer phenotype functions at least in part in competitive interactions, particularly with other species, perhaps different micro-environments have different competitors, selecting for different killers. Or competitors might evolve resistance to common toxins, giving advantage to the rare types, thus maintaining diversity.
(5) Host–symbiont adaptations. Whether the dsRNAs are ultimately good or harmful to their hosts, their interests are unlikely to be completely aligned, and so, there may be selection on the hosts at times to suppress them and to the extent that these defences are satellite-specific, then there may be a rare type advantage, promoting diversity. More generally, there are many host factors that affect viral replication (e.g. SKI and MAK genes; Wickner 1974, Ball et al. 1984, Ridley et al. 1984, Wickner 1996b, Magliani et al. 1997), and evolution in these (for any reason) could have pleiotropic effects on the type of dsRNA that is maintained in the cell.
(6) Virus–satellite interactions. While in our sample, all L-A viruses were accompanied by a satellite, this observation does not in itself resolve whether the interaction between them is mutualistic or parasitic (which may depend on whether the satellite is a killer or not), and, even if mutualistic, again, their interests may not be completely aligned, and conceivably antagonistic coevolution between them may select for a diversity of satellites.
(7) The selectively neutral null hypothesis. The frequency and distribution of the different viruses reflect a balance between mutation, horizontal transmission, and drift in spatially structured environments (Charlesworth and Charlesworth 2010). Each of these hypotheses is open to empirical testing.

As the M and L dsRNAs are replicated by the same polymerase, the null expectation is that they would have the same rate of mutation and evolution, but the contrast between the L-As (which are alignable across all strains) and the Ms (which are unalignable among types, perhaps because they have completely different origins) makes comparisons of evolutionary rates difficult. One comparison that can be made is between the M and L sequences of our T21.4 strain and those from the Lithuanian AML-15–66 strain (Vepštaitė-Monstavičė et al. 2018). In this case, Ms are alignable, and the sequence divergence in coding regions was not dramatically different from that in L-As (23% versus 30% for 4-fold degenerate sites and 3.5% versus 1.2% at 0-fold sites, for M and L-A respectively, giving an overall divergence of 10% for both dsRNAs).

More generally, while different M types were unalignable, there was little sequence variation within M types, apart from the potential inversion polymorphism and the ragged 5′ ends. M21 and M74 had SNP variants scattered throughout the sequence that, together with the inversion, created five variants of M21 and three variants of M74 (Table 1). Some infectious RNA viruses have notoriously high mutation rates, leading to the notion of ‘quasi-species’ (Domingo and Perales 2019), but there was no sign of this in our data. A similar result was reported for variant Ms in *S. cerevisiae* (Ramírez et al. 2017), and the authors proposed there may be a recombination-mediated dsRNA repair processes maintaining homogeneity. Alternatively, we suggest there has been much less selection for rapid replication rates in these predominantly vertically transmitted dsRNAs compared to infectious viruses, and that has allowed the L-A-encoded RNA polymerase to evolve a higher fidelity. It would be interesting to directly measure the error rate.

Our study has revealed a surprisingly high diversity of dsRNA M satellites in a natural *S. paradoxus* population in the UK, comprising six distinct types, alongside very limited diversity within types (except perhaps at the terminal regions) and limited diversity of their associated L-A viruses. Three of these M satellites are newly identified and share some key structural features with previously described Ms at both nucleotide and amino acid levels. Both M and L-A dsRNAs share similar structural features at the 5′ ends, presumably reflecting their common dependency on the same replication and encapsidation system encoded by L-A and the same host translation machinery. The coexistence of multiple, highly divergent potentially toxic M satellites within a single population of yeast remains unexplained and may reflect a combination of ecological interactions, host–virus conflict resolutions, and population-level processes.

## Supplementary Material

Supplementary_Figures_and_Tables-FV_veag043

Appendix_veag043

## Data Availability

All sequences were generated in this study have been deposited in GenBank under the accession numbers listed below: L-A-62.1 (PX516181), L-A-62.2 (PX516182), L-A-21.1 (PX516183), L-A-21.2 (PX516184), L-A-74.1 (PX516185) L-A-74.2 (PX516186), L-A-SW1.1 (PX516187), L-A-SW1.2 (PX516188), L-A-SW3.1 (PX516189), L-A-SW3.2 (PX516190), L-A-SW4.2 (PX516191), L-A-SW5.1 (PX516192), L-A-SW5.2 (PX516193), L-A-SW6.1 (PX516194), L-A-SW6.2 (PX516195), M62 (PX516196), M62-SW (PX516197), M21 (PX516198), M21-SW1 (PX516199), M21-SW2 (PX516200), M21-SW3 (PX516201), M21-SW4 (PX516202), M74 (PX516203), M74-SW1 (PX516204), M74-SW2 (PX516205), M-SW1.1 (PX516206), M-SW1.2 (PX516207), M-SW2.1 (PX516208), M-SW2.2 (PX516209), M-SW3.1 (PX516210), M-SW3.2 (PX516211), M74-APA1 (PX516212), M74-APA2 (PX516213), APA1 (PX516214), and APA2 (PX516215).
