# Supplementary material for: Genomic characterization and diversity of dsRNA viruses and their killer satellites in a natural population of the yeast Saccharomyces paradoxus
Source: Virus Evol. 2026 Jul 16;12(1):veag043. doi: 10.1093/ve/veag043 (PMC13449100; doi:10.1093/ve/veag043)
Supplement: Supplementary file 1 [file appendix_veag043.docx]

Appendix 1. Results of the phenotypic survey for killer activity, screens for dsRNAs, S1 nuclease treatment, and cycloheximide treatment. Strains in bold are those sequenced in pool. √: Positive; --: not observed; blank: not tested

| **Strain Name** | **Genome¹** | **Year collected** | **Killer assay test** | | | **dsRNA present on agarose gel** | | **S1 nuclease treatment ³** | **Cycloheximide treatment** ⁴ | | |
| --- | --- | --- | --- | --- | --- | --- | --- | --- | --- | --- | --- |
|  |  |  | This study² | Chang, Leu, & Chang, 2015 | Pieczynska, de Visser, & Korona, 2013 | L (4.5 Kb) | M (1-2.5 kb) |  | L | M | Killer assay ⁵ |
| **Q14.4** |  | 1998 | K |  |  | √ | √ | √ | √ | -- | NK |
| Q15.1 |  | 1998 | NK |  |  |  |  |  |  |  |  |
| **Q16.1** |  | 1998 | NK |  |  | √ | √ | √ | √ | -- | NK |
| Q31.4 | G2 | 1998 | NK | NK | NK |  |  |  |  |  |  |
| Q32.3 |  | 1998 | NK | NK | NK |  |  |  |  |  |  |
| **Q43.5** |  | 1998 | K |  |  | √ | √ | √ | √ | -- | NK |
| **Q59.1** |  | 1998 | K | NK | NK | √ | √ | √ | √ | -- | NK |
| Q6.1 |  | 1998 | NK |  |  | -- | -- |  |  |  |  |
| **Q62.5** |  | 1998 | K | K | K | √ | √ | √ | √ | -- | NK |
| Q69.8 |  | 1998 | NK | NK | NK |  |  |  |  |  |  |
| Q70.8 |  | 1998 | NK |  |  |  |  |  |  |  |  |
| **Q74.4** |  | 1998 | K | K | K | √ | √ | √ | √ | -- | NK |
| Q89.8 |  | 1998 | NK | NK | NK |  |  |  |  |  |  |
| **Q95.3** |  | 1998 | NK | NK | NK | √ | √ | PD | √ | -- | NK |
| S36.7 |  | 1997 | NK | NK | NK | -- | -- |  |  |  |  |
| T18.2 |  | 1998 | NK |  |  | -- | -- |  |  |  |  |
| **T21.4** | G3 | 1998 | K | K | K | √ | √ |  | √ | -- | NK |
| T22.1 |  | 1998 | K |  |  |  |  |  |  |  |  |
| **T26.3** |  | 1998 | NK |  |  | √ | √ | √ | √ | -- | NK |
| T27.3 |  | 1998 | K |  |  |  |  |  |  |  |  |
| T32.1 |  | 1998 | K |  |  |  |  |  |  |  |  |
| **T4b** |  | 1998 | K |  |  | √ | √ |  | √ | -- | NK |
| T62.1 |  | 1998 | NK |  |  |  |  |  |  |  |  |
| **T68.2** |  | 1998 | K |  |  | √ | √ | √ | √ | -- | NK |
| **T68.3** |  | 1998 | K |  |  | √ | √ | √ | √ | -- | NK |
| T74.4 |  | 1998 | K |  |  |  |  |  |  |  |  |
| T76.2 |  | 1998 | NK |  |  | -- | -- |  |  |  |  |
| T76.6 | G2 | 1998 | NK |  |  | -- | -- |  |  |  |  |
| **T8.1** | G3 | 1998 | K |  |  | √ | √ |  | √ | -- | NK |
| W7 |  | 1996 | NK | NK | NK | -- | -- |  |  |  |  |
| **Y1** |  | 2004 | NK |  |  | √ | √ | PD |  | -- | NK |
| **Y10** |  | 2004 | K |  |  | √ | √ |  | √ | -- | NK |
| Y2 |  | 2004 | NK |  |  |  |  |  |  |  |  |
| **Y2.2** |  | 2004 | NK |  |  |  |  |  |  |  |  |
| Y2.6 |  | 2004 | NK |  |  | -- | -- |  |  |  |  |
| Y2.8 |  | 2004 | NK |  |  | √ | √ | -- | √ | -- | NK |
| Y3 |  | 2004 | NK |  |  |  |  |  |  |  |  |
| Y4 |  | 2004 | NK |  |  |  |  |  |  |  |  |
| Y4.5 |  | 2004 | NK |  |  |  |  |  |  |  |  |
| Y5 |  | 2004 | NK |  |  |  |  |  |  |  |  |
| Y5.1 |  | 2004 | NK |  |  |  |  |  |  |  |  |
| Y5.6 |  | 2004 | NK |  |  |  |  |  |  |  |  |
| Y5.8 |  | 2004 | NK |  |  |  |  |  |  |  |  |
| Y6 |  | 2004 | NK |  |  |  |  |  |  |  |  |
| Y6.2 |  | 2004 | NK |  |  |  |  |  |  |  |  |
| Y6.5 | G1 | 2004 | NK | NK | NK | -- | -- |  |  |  |  |
| Y7 |  | 2004 | NK | NK | NK | -- | -- |  |  |  |  |
| Y7.1 |  | 2004 | NK |  |  |  |  |  |  |  |  |
| Y7.5 |  | 2004 | NK |  |  |  |  |  |  |  |  |
| Y8 |  | 2004 | NK |  |  |  |  |  |  |  |  |
| Y8.1 |  | 2004 | NK | NK | NK |  |  |  |  |  |  |
| Y8.4 |  | 2004 | NK |  |  |  |  |  |  |  |  |
| **Y8.5** |  | 2004 | K | K | K | √ | √ | √ | √ | -- | NK |
| Y8.6 |  | 2004 | NK |  |  |  |  |  |  |  |  |
| Y8.8 |  | 2004 | NK |  |  |  |  |  |  |  |  |
| Y9 |  | 2004 | NK |  |  |  |  |  |  |  |  |
| Y9.6 |  | 2004 | NK | NK | NK | -- | -- | -- |  |  |  |
| Z1 |  | 2004 | NK | NK |  |  |  |  |  |  |  |
| Z1.1 |  | 2004 | NK | NK | NK | -- | -- | -- |  |  |  |
| Z3 | G1 | 2004 | NK |  |  | -- | -- | -- |  |  |  |

¹ Indicates strains with genomes sequenced; G1, G2 and G3 show pairs with identical genomes.

² This column indicates the outcomes of both same-day and two-day assays, identifying the sample as either Killer (K) or non-killer (NK).

³ S1 nuclease treatment: S1 Nuclease cuts most of the M dsRNAs in the middle when the treatment is performed at 67°C. √ indicates complete digestion, PD indicates partial digestion, and – indicates no digestion occurred.

⁴ Cycloheximide treatment removes M dsRNA from the infected strains.

⁵A killer assay was done following the cycloheximide treatment to confirm killer activity was gone.
